# Evaluating the Potential for the Environmentally Sustainable Control of Foot and Mouth Disease in Sub-Saharan Africa

**DOI:** 10.1007/s10393-013-0850-6

**Published:** 2013-06-25

**Authors:** Kenneth J. Ferguson, Sarah Cleaveland, Daniel Thomas Haydon, Alexandre Caron, Richard A. Kock, Tiziana Lembo, J. Grant C. Hopcraft, Bertrand Chardonnet, Thomas Nyariki, Julius Keyyu, David James Paton, Fredrick Mathias Kivaria

**Affiliations:** 1 Institute of Biodiversity, Animal Health and Comparative Medicine, College of Medical, Veterinary and Life Sciences, University of Glasgow, Glasgow, G12 8QQ Scotland, UK; 2 Department of Environment and Societies, CIRAD-UPR AGIRs, P.O. Box 1378, Harare, Zimbabwe; 3 Department of Environment and Societies, CIRAD, UPR AGIRs, Montpellier, France; 4 Mammal Research Institute, University of Pretoria, Pretoria, South Africa; 5 Department of Pathology & Infectious Diseases, The Royal Veterinary College, Hatfield, Hertfordshire AL9 7TA UK; 6 African Protected Areas & Wildlife Expert, 92210 Saint Cloud, France; 7 African Union Inter-African Bureau for Animal Resources, P.O. Box 30786, Nairobi, Kenya; 8 Tanzania Wildlife Research Institute, P.O. Box 661, Arusha, Tanzania; 9 The Pirbright Institute, Pirbright, Surrey GU24 0NF UK; 10 Ministry of Livestock Development and Fisheries, P.O. Box 9152, Dar es Salaam, Tanzania

**Keywords:** foot-and-mouth disease, Africa, control, FAO-OIE, sustainable, environment, wildlife, integration, decision-making

## Abstract

Strategies to control transboundary diseases have in the past generated unintended negative consequences for both the environment and local human populations. Integrating perspectives from across disciplines, including livestock, veterinary and conservation sectors, is necessary for identifying disease control strategies that optimise environmental goods and services at the wildlife-livestock interface. Prompted by the recent development of a global strategy for the control and elimination of foot-and-mouth disease (FMD), this paper seeks insight into the consequences of, and rational options for potential FMD control measures in relation to environmental, conservation and human poverty considerations in Africa. We suggest a more environmentally nuanced process of FMD control that safe-guards the integrity of wild populations and the ecosystem dynamics on which human livelihoods depend while simultaneously improving socio-economic conditions of rural people. In particular, we outline five major issues that need to be considered: 1) improved understanding of the different FMD viral strains and how they circulate between domestic and wildlife populations; 2) an appreciation for the economic value of wildlife for many African countries whose presence might preclude the country from ever achieving an FMD-free status; 3) exploring ways in which livestock production can be improved without compromising wildlife such as implementing commodity-based trading schemes; 4) introducing a participatory approach involving local farmers and the national veterinary services in the control of FMD; and 5) finally the possibility that transfrontier conservation might offer new hope of integrating decision-making at the wildlife-livestock interface.

## Introduction: The Progressive Control Pathway for FMD Control

In June of 2012 multi-lateral agencies including the Food and Agriculture Organisation (FAO), the World Animal Health Organisation (OIE) and the World Bank joined with national stakeholders, in Bangkok Thailand, to endorse a global strategy that aims to ‘progressively reduce the impact of foot-and-mouth disease (FMD) and the load of FMD virus’ (FAO-OIE 2012). Following the successful world-wide campaign to eradicate rinderpest, animal health professionals now believe that a further concerted global effort to control, or, in some cases eradicate FMD is a goal worth planning. FMD is the most economically damaging transboundary livestock disease worldwide and its control would also have potential to benefit the poorest livestock-keepers (Kivaria 2003; Perry and Grace 2009).

**Figure 1:**
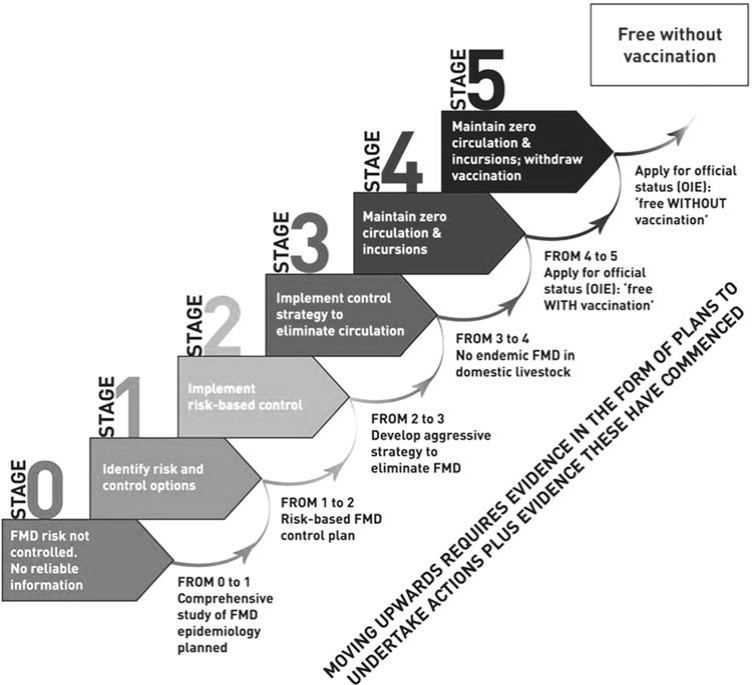
The stages of the PCP-FMD (courtesy of FAO-OIE 2012).

The strategy that is set to achieve this aim is termed the Progressive Control Pathway for FMD (PCP-FMD, FAO-OIE 2012) which lays out a 5-stage plan to guide regional and national authorities in developing risk-based FMD control strategies based on a clear set of activities and progression stages. The steps span from stage 0, a situation where there is no information about FMD and no control measures in place, to stage 5, which represents freedom from infection (Fig. 1).

The PCP-FMD recognises that key outcomes and objectives of FMD control, and the approaches used to achieve this, are likely to vary in different countries. Progress to stage 5 may not always be possible given different agro-ecological systems. Unexpected consequences of the measures taken to reach stage 5 may outweigh the benefits of doing so. Therefore, it is an important principle that each stage should be considered a benefit in itself and worth pursuing irrespective of whether stage 5 can or will ever be reached. The range of potential scenarios and optimum strategies is particularly diverse in Africa, reflecting both the epidemiological complexity of FMD on the continent, which involves cycles of infection in wildlife, and the potentially wide range of beneficiaries of FMD control, who include subsistence livestock-keepers, commercial farmers and national economies. For example, in addition to developing opportunities for livestock trade, an important objective of FMD control in Africa relates to poverty alleviation and enhancing food security in the traditional pastoral sector, which provides the source of the vast majority of milk and meat consumed (Perry and Grace 2009). While FMD has often been regarded as an insignificant disease in extensive systems, there is clear evidence that losses from reduced production and market access can be substantial (Kivaria 2003; Perry and Grace 2009, FAO-OIE 2012, Lembo et al. 2012) and that control of FMD may have widespread benefits.

In this paper, we address a suite of interacting issues in relation to implementation of the PCP-FMD in Africa, highlighting the importance of engagement between the livestock and wildlife sectors in considering potential FMD control strategies. We further discuss how control options might be developed that deliver a range of international to local economic and development benefits, while protecting the continent’s wildlife and natural resources.

## Discussion: Key Actions for an Integrated Approach to FMD Control

FMD circulation in domestic and wild populations in Africa is characterised by a complex epidemiology involving multiple serotypes and topotypes that naturally circulate in a range of species (Thomson et al. 2003; Bronsvoort et al. 2008). Wildlife species, particularly African buffalo (*Syncerus caffer*), can harbour indigenous SAT (Southern African Type) strains of FMD, and act as sources of infection and outbreaks in livestock (Bengis 2005; Vosloo et al. 2002). To protect the FMD-free status of livestock and comply with stringent zoosanitary regulations demanded for export of livestock and livestock products (FAO-OIE 2012), some countries in southern Africa have, in the past, adopted disease control strategies to prevent: (1) viral transmission between buffalo and livestock, such as veterinary cordon fencing around wildlife-protected areas and removal of wildlife from outside-protected areas and (2) viral transmission from high risk cattle populations living in the periphery of protected areas to naïve cattle populations further away using vaccination and movement control zones. However, there is substantial evidence that veterinary cordon fencing, which has effectively led to geographic zonation and restriction of wildlife movements, has been highly detrimental to wildlife populations and can modify animal community structure (Williamson and Williamson 1981; Taylor and Martin 1987; Harris et al. 2009; Ferguson and Hanks 2010, 2012; Gadd 2011; Scoones et al. 2010; Cozzi et al. 2013). Furthermore, movement control zones have also negatively affected farmers by limiting trading opportunities to the detriment of their livestock production (McGahey 2011). Within the arid and semi-arid rangelands of Africa, livestock movements are essential for the viability of pastoral systems. Any restriction of movements could compromise the overall economic and environmental benefits of pastoral production systems, which can yield greater economic benefits in comparison with commercial fenced ranch systems and arguably causes less environmental damage and has landscape restorative potential (Boone 2007; de Leeuw and Peacock 1982; McGranaghan 2008; Lindsey et al. 2013).

While southern African countries have focused on the risk posed by buffalo, the contribution of buffalo to FMD outbreaks in livestock in other parts of Africa remains unclear and the role of other wild ungulate species is still debated (Hargreaves et al. 2004; Kock et al., in press). It is hoped that activities identified within the PCP-FMD will allow more complete evaluation of the risks associated with wildlife, providing opportunities for risk-based approaches that are more environmentally nuanced and allow for the development of disease control pathways that address widespread concerns about the impact of FMD control on wildlife conservation (FAO-OIE 2012; Kock et al. 2006).

There has been increasing collaboration between the conservation and livestock sectors as a result of growing recognition for the need for emphasizing environmentally-sensitive approaches (D’Amico Hales et al. 2004; Osofsky et al. 2005, 2011), especially with regard to the management of transfrontier conservation areas in southern Africa. However, there is still significant room for improvement in terms of the joint development of FMD control strategies, highlighted by the fact that few conservationists are aware of the PCP-FMD or its implications for management of wildlife conservation areas.

From the additional perspectives of livestock development, as it relates to poverty alleviation, the PCP-FMD also needs to ensure that benefits of disease control filter down to local farmers. However, the importance of FMD as an economic driver of poverty is likely to vary in different agro-ecological and livestock production systems. Livestock production at the periphery of protected areas is often low for many reasons, including husbandry practices, pasture quality and infectious diseases, some of which are shared with wildlife (Caron et al. 2013). Although pastoralists in East Africa indicate that, after tick-borne diseases, FMD is among the most important of livestock diseases (Cleaveland et al. 2001; Bedelian et al. 2007; Ohaga et al. 2007; Jost et al. 2010; De Garine-Wichatitsky et al. 2013; Catley et al. 2013) this may not apply in other regions and other farming systems. Furthermore, the potential uptake by farmers of control strategies, such as FMD vaccination (although efficacious vaccines and delivery remain elusive) is uncertain. Therefore the imperative for FMD control in livestock, and the balance of costs and benefits of disease control at the wildlife–livestock interface are likely to vary for different communities and for different ecological regions in Africa.

The advent of integrated multiple-land use policies encapsulated by the strategic vision of ‘transfrontier conservation’ within a One Health framework (Hanks 2003; Osofsky et al. 2008) represents a major paradigm shift and step forward in African conservation. While these large tracts of African land earmarked for a mixed conservation and development agenda have enormous potential in connecting wildlife populations, and enhancing the integrity and viability of protected areas, they also create substantial inter-sectoral challenges, with transboundary diseases causing potential flashpoints (Bengis 2005; De Garine-Wichatitsky et al. 2012). Many multiple-use areas straddle international borders, often in regions of high FMD endemism. In such overlapping situations there is an urgent need to develop coordinated and integrated land use planning strategies to manage livestock production, a key livelihood option in these semi-arid areas, whilst simultaneously protecting the environment (Karesh et al. 2002; Kivaria 2003; Rweyemamu et al. 2012; Murwira et al. 2012).

Part of the challenge relates to assumptions about potential threats associated with livestock–wildlife interactions. While diseases, such as FMD, can be transmitted from wildlife to livestock, risk-based strategies can be adopted to manage disease risk without harm to wildlife populations. Conversely, livestock populations do not invariably pose a threat to wildlife, and can have synergistic interactions, including diversifying livelihood options for local communities, promoting localised increases in biodiversity and serving as a land-use bulwark against increased savanna conversion to cropping and mechanized agriculture (Reid 2012; Riginos et al. 2012).

Joint development of environmentally sensitive control measures for FMD provides a valuable opportunity to broaden perspectives across sectors and promote awareness of the shared benefits of disease control at the livestock–wildlife interface. The PCP-FMD can act as a potential framework for this, but, to be successful, FMD policy development needs to be aligned with conservation and socioeconomic factors at the initial phases of the planning process. The PCP-FMD requires the identification of specific environmental standards and objective core activities to be conducted early in the stage process and from a transdisciplinary sectoral perspective (Karesh et al. 2002; Rweyemamu et al. 2012) not as an ‘add-on’ some way down the roadmap.

What needs to be done to ensure that interventions to control FMD are as environmentally sensitive and locally acceptable as possible? Several options are currently available as part of a strategy ‘tool box’ that may be considered by participating PCP-FMD countries, especially those with large wildlife populations.

First, improved understanding of circulating viral strains and risk factors provides opportunities for exploring livestock vaccination strategies. For example, growing evidence from both West and East Africa indicates that livestock factors, including cattle movements, are major drivers of endemic FMD (Kivaria 2003; Bronsvoort et al. 2004; Picado et al. 2011), and that proximity to wildlife-protected areas is not consistently identified as a risk factor for livestock outbreaks (Picado et al. 2011; Lembo et al. 2012). Conversely, in southern Africa, recent studies indicate that interactions between buffalo and cattle can account for FMD primary outbreaks at wildlife/livestock interface (Miguel et al. 2013; De Garine-Wichatitsky et al. 2012). Knowledge of the diversity and circulation patterns of different strains of FMD in different hosts (i.e. wildlife and small ruminants) across Africa is also growing, allowing vaccine strains to be selected that can be tailored to local settings, increasing their likelihood of being more effective.

Second, freedom from disease and infection (stages 4 and 5 of the PCP-FMD), which has been required as part of international sanitary regulations for the global export of livestock and livestock products, but is precluded by the co-existence of livestock and wildlife, may not be an appropriate or realistic objective for many African countries. Even where development of trade opportunities remains a key objective, freedom from disease may be unnecessary given the opportunities for developing markets through commodity-based trade and less-restrictive regional trade (Scoones et al. 2010). Commodity-based trade, representing a value chain approach and a wide range of possible value-added, processed products, is a promising model for decreasing the risk of FMD infection in processed animal protein, which meets internationally recognised standards to ensure the lowest risk possible (Thomson et al. 2004; Paton et al. 2010). Strategies incorporating commodity-based trading could re-balance the need for the safe trade of beef products on the one hand whilst also re-aligning appropriate conservation initiatives with agriculture programmes (Thomson et al. 2004; Cumming 2010; Scoones et al. 2010).

Third, in wildlife-rich countries, livestock production areas could still be identified for more commercial development, for example selecting areas with natural barriers, such as mountain ranges and lakes, that would achieve natural separation of buffalo and livestock. The concept of disease control based around geographical zones and compartments will likely remain critical to the control process, and, for these strategies, improved vaccines and controls on livestock movements remain the key fundamentals to FMD control. Therefore, separating livestock, that live well away from wildlife enclaves and other FMD risks may well be required for a successful implementation of the PCP-FMD. Compartments in which management practices (such as bio-security and vaccination informed by adequate knowledge of surrounding risk, with or without geographic isolation) keep smaller enclaves of livestock disease-free may also represent a possible means for contributing to exports from countries that cannot attain complete disease freedom.

Fourth, in order to be more easily accepted and negotiated locally, FMD control at the wildlife/livestock interface should be included in a veterinary “service delivery package”, with communities involved in discussions with veterinary services to identify and implement important disease control objectives. Such a participatory approach could lead to a more efficiently implemented PCP-FMD, and avoid possible negative perceptions that may arise from veterinary services imposing FMD disease control on local communities driven primarily by the benefits of national production and trade. FMD control is not necessarily the first priority of most local farmers. However, farmers expect greater interventions by the veterinary services on specific diseases that impact heavily on their production efforts and therefore directly on their livelihoods. If veterinary services, often (negatively) perceived as representing the national authority in these remote areas, could enter into a negotiation process with communities, they could agree and parcel out their respective responsibilities in achieving FMD and other important disease control objectives. This participatory action could lead to a more efficiently implemented PCP-FMD. A parallel negotiation process involving conservation authorities and local communities could similarly provide a framework to manage and mitigate wildlife/livestock interactions, for example by managing livestock access to water points during the dry season or by developing adapted grazing strategies for livestock that aim to limit cross-species contact.

Finally, the advent of Transfrontier conservation is predicated on a ‘mixed’ economy of wildlife and agriculture. The land-use planning process that attempts to marry these two sectors, especially where they overlap at the peripheries of protected areas, makes much use of the current decentralization dispensation which pushes for the devolution to the subsidiary level (community) of natural resource user rights and the expansion of ecotourism/community conservancies in many key biodiversity locations. These rights and processes are setting up new and complex interfaces between wildlife and livestock, which are often governed locally.

We can conclude our toolbox outline by recognising that mobility and connectivity of species and landscape elements are critically shared fulcra of livestock development and conservation advancement in savanna Africa.

The above strategies help to emphasize that, in an increasingly globalized world, animal health professionals are dealing with a highly mobile interface between wildlife, livestock, and pathogens and that this reality must serve as the foundation stone of ameliorative actions.

If we examine the role of the PCP-FMD in an East African context we find that the region still has growing livestock populations co-mingling with large, but generally declining wildlife populations (Craigie et al. 2010). Furthermore, despite no history of veterinary fencing, this region does face significant challenges from landscape fragmentation and degradation, and losses of wildlife populations, linked in many cases to agricultural management and practices (Newmark 2008; Alkemade et al. 2012; Reid 2012). How might the PCP-FMD assist in changing this trajectory?

Tanzania harbours half of Africa’s buffalo population and the third largest cattle population in Africa (IUCN 2012). The wildlife sector is critical to Tanzania’s economy, with wildlife tourism contributing 8% of GDP in 2010. Several wildlife-protected ecosystems in Tanzania are recognised as World Heritage Sites of global importance and have been traditionally managed in line with low-intervention policies. However, there is also enormous potential for developing the livestock sector, with increasing demand for meat from rapidly growing urban populations in Tanzania and elsewhere. The Ministry of Livestock and Fisheries Development has expressed a commitment to progress from stages 0 to 3 of the PCP-FMD by 2020, within a broader livestock policy that has a stated objective of supporting livestock development while conserving the environment (URT 2009; Lugoe 2011; FAO-OIE 2012). We propose that this may be achieved by a combination of the above five approaches outlined in this paper.

Much depends on the outcome of current research, which aims to characterise the diversity of viruses to inform the selection of locally-appropriate vaccines, and to understand transmission patterns among livestock, buffalo and other potential wild and domestic host populations (Paton et al. 2009). With improved vaccines, livestock vaccination can be deployed, with strategies designed to mitigate disease impacts for individual farmers as well as to disrupt virus circulation across larger scales, for example, by ensuring high vaccine coverage against SAT viruses on the borders of protected areas. With some control of disease achieved through livestock vaccination, greater integration of rangeland uses, through separation strategies could still be important at local levels, which may have benefits for both wildlife and livestock (Reid et al. 2008; Reid 2012; Riginos et al. 2012). For example, a ‘mixed wild and domestic species’ farming model adopted in Laikipia (Kenya) has been successful and sustainable in terms of conserving the environment and generating tourist income (Augustine et al. 2011; Reid et al. 2008). Tanzania has long been at the forefront of multiple land-use initiatives, for example the establishment of the Ngorongoro Conservation Area in 1959, and Tanzania’s rangelands are still large and diverse in terms of spectacular wildlife and traditional pastoralism. However, both will come under serious threat unless the agriculture and conservation sectors can work effectively and efficiently together (Kock et al. 2010). This will require a high degree of policy harmonization, by dovetailing global and national FMD control policies with global, national and regional biodiversity conservation strategies with the ultimate result being FMD control methods aligned to an objective of a disease-free and wildlife-friendly environment.

## Conclusions and Signs of Progress

African conservation is competing in an ever more globalized world, but many aspects of conservation such as mixed management of livestock and wildlife are hampered by strict ‘freedom from disease’ policies. Could the PCP-FMD, with its emphasis on flexibility and regionalism, lead by example and factor in wildlife and ecosystem resources within an overall social and economic development policy, which is also appropriate to the biodiverse and yet increasingly fragmented rangelands of Africa? We think it could. However, in the long-term FMD control may prove counter-productive if it impacts negatively on other key factors necessary to achieve ‘healthy landscapes’, such as the perception of local communities towards wildlife conservation, or if it promotes unsustainable livestock production systems. We argue that balanced and integrated (top down and bottom up) land use planning is essential to the health of both the conservation and agricultural sectors. In particular the concept of an environmentally sensitive and locally focussed approach to disease control may encourage greater interaction between decision-making policies for agricultural development and conservation that draws on all available evidence and expertise, including an integrated, scientific risk assessment of multiple livestock diseases and their control in relation to conservation (Mariner et al. 2012).

There are signs of progress in terms of integrating conservation and livestock disease control decision-making. The Southern African Development Community Livestock Technical Committee has recently acknowledged the potential of alternative options for local cattle production (i.e., commodity-based trade—see the Phakalane Declaration—http://www.wcs-ahead.org/phakalane_declaration.html) paving the way to offering local producers a way of benefiting more from livestock production even on the periphery of protected areas. The PCP-FMD pathway will benefit in this region from more communication between authorities and local stakeholders and regional collaboration to manage the disease. On a broader front an attempt at mainstreaming environmental issues with development goals is the rationale behind Tanzania’s MKUKUTA (National Strategy for Growth and Reduction of Poverty) process. This highly participatory planning model could serve as a platform for the efficient integration of environmentally sustainable livestock disease control and wildlife linked land use policies in that country (Swiderska and Maganga 2008; URT 2009).

We urge that the PCP-FMD implementing process should start to involve multiple stakeholders, including conservationists, and representatives of local communities, who can collectively place emphasis on environmental and disease risk assessments, at both national and regional levels such that environmental standards and impact assessments become the accepted norm for all disease control policies that may impact on Africa’s wildlife heritage.
